# Strain Rate-Dependent Compressive Properties of Bulk Cylindrical 3D-Printed Samples from 316L Stainless Steel

**DOI:** 10.3390/ma15030941

**Published:** 2022-01-26

**Authors:** Michaela Neuhäuserová, Petr Koudelka, Tomáš Fíla, Jan Falta, Václav Rada, Jan Šleichrt, Petr Zlámal, Anja Mauko, Ondřej Jiroušek

**Affiliations:** 1 Department of Mechanics and Materials, Faculty of Transportation Sciences, Czech Technical University in Prague, Na Florenci 25, 110 00 Prague 1, Czech Republic; koudelka@fd.cvut.cz (P.K.); fila@fd.cvut.cz (T.F.); faltaja2@fd.cvut.cz (J.F.); sleichrt@fd.cvut.cz (J.Š.); zlamal@fd.cvut.cz (P.Z.); jirousek@fd.cvut.cz (O.J.); 2 Czech Academy of Sciences, Institute of Theoretical and Applied Mechanics, Prosecká 809/76, 190 00 Prague 9, Czech Republic; rada@fd.cvut.cz; 3 Faculty of Mechanical Engineering, University of Maribor, Smetanova ul. 17, 2000 Maribor, Slovenia; anja.mauko@um.si

**Keywords:** 3D printing, laser powder bed fusion, 316L stainless steel, printing direction, split Hopkinson pressure bar

## Abstract

The main aim of the study was to analyse the strain rate sensitivity of the compressive deformation response in bulk 3D-printed samples from 316L stainless steel according to the printing orientation. The laser powder bed fusion (LPBF) method of metal additive manufacturing was utilised for the production of the samples with three different printing orientations: 0${}^{\circ }$, 45${}^{\circ }$, and 90${}^{\circ }$. The specimens were experimentally investigated during uni-axial quasi-static and dynamic loading. A split Hopkinson pressure bar (SHPB) apparatus was used for the dynamic experiments. The experiments were observed using a high-resolution (quasi-static loading) or a high-speed visible-light camera and a high-speed thermographic camera (dynamic loading) to allow for the quantitative and qualitative analysis of the deformation processes. Digital image correlation (DIC) software was used for the evaluation of displacement fields. To assess the deformation behaviour of the 3D-printed bulk samples and strain rate related properties, an analysis of the true stress–true strain diagrams from quasi-static and dynamic experiments as well as the thermograms captured during the dynamic loading was performed. The results revealed a strong strain rate effect on the mechanical response of the investigated material. Furthermore, a dependency of the strain-rate sensitivity on the printing orientation was identified.

## 1. Introduction

Three-dimensional printing/additive manufacturing (AM) has become a competitive production method for various kinds of materials and applications, first and foremost due to the principle of successively adding material based on a CAD model of the produced part [1]. This allows for the fabrication of structures with complex geometries that would be difficult or even impossible to produce using conventional manufacturing methods [2]. Generally, AM refers to several different techniques that, according to the International Organisation for Standardization (ISO), may be classified into seven categories: (a) binder jetting (BJ), (b) directed energy deposition (DED), (c) material extrusion (ME), (d) material jetting (MJ), (e) powder bed fusion (PBF), (f) sheet lamination (SL), and (g) vat photo-polymerisation (VP) [1]. However, only specific techniques are suitable for a specific type of material. In the case of metals or composite materials containing metal particles, the PBF technique is the most commonly used method in metal AM processes, even though any of the listed technologies may be used. The basic principle of the PBF method is the layer-by-layer melting or sintering of a powdered metal using a high-power heat source such as laser or electron beam [3,4,5].

The most common materials used for metal AM are steel, aluminum alloys, titanium and its alloys, nickel-based alloys, copper, or cobalt chrome. However, steel is still the most frequently used engineering material [6] and the steel grades suitable for AM include 316L [4,7,8] and 304L [9,10] stainless steels, maraging steels [4], and precipitation hardening stainless steels [11]. Particularly, the AM of 316L stainless steel has attracted attention due to the superior properties of this stainless steel grade, such as high toughness or ductility compared with other stainless steels, making it suitable for applications in the biomedical, automotive, or aerospace industries [4,12,13]. However, to utilise 3D-printed structures in safety-critical applications, a reliable numerical analysis of the problem must precede the manufacturing process. Here, the properties of the 3D-printed material need to be described with reasonable precision and reliability on a broad scale of loading conditions to enable the prediction of the mechanical behaviour of the structural parts in real applications [14].

One of the interesting applications of metal AM methods is the production of lattices for structural applications, such as deformation energy mitigation and ballistic protection, where auxetic lattices have attracted considerable attention. In this field, the strain rate-dependency of such porous solids is given by the architecture of the lattice and the base material used for its production. Here, we have already shown that a split Hopkinson pressure bar (SHPB) instrumented with a high-speed camera can be used to assess the dynamic compressive response of various auxetic lattices manufactured by LPBF from 316L powdered austenitic steel [15,16,17]. The SHPB apparatus has also been used to evaluate the influence of elevated and reduced temperatures on the deformation response of steel auxetic lattices [18]. Furthermore, we have used modified Hopkinson pressure bar devices to investigate the dynamic impact characteristics of lattice structures [19] and metal foam-based lightweight cellular solids [20], where the possibility to use ex situ X-ray tomography has also been demonstrated.

Generally, metal AM methods are well known for large sets of process parameters influencing the resulting constructs that include, e.g., the scanning strategy, scanning speed, hatching distance, metal powder granularity, powder layer thickness, laser power, etc. [7]. The effect of the energy density (combined parameter of laser power, hatch distance, scan speed and layer thickness) on the microstructure of the 3D-printed 316L stainless steel and its tensile strength was described in [21]. Studies analysing the effect of the energy density and scanning strategy are also available in the literature [22,23]. Furthermore, the design of the part being produced plays an important role in the structure and properties of the 3D-printed material itself. Here, the size effect of the produced part on the material microstructure and mechanical behaviour was presented in [24,25] and the influence of the geometrical orientation of the 3D-printed part on the mechanical properties and micro-structure of the material was described in [26]. The literature review indicates that metal AM is a multi parameter problem and the quality of the 3D-printed material may be affected by several factors. Sing et al. [27] suggest machine learning as a viable method for process parameter optimisation and as a competitive tool compared to expensive and demanding experimental studies.

However, experimental investigations are necessary in order to fully understand the processes occurring in the material during loading. Additionally, to be able to predict the deformation responsse of AM lattices using both analytical models and numerical simulations, it is necessary to assess the relevant mechanical characteristics of the specific base material used in the AM production process. The present study is focused on an experimental investigation of the compressive mechanical characteristics of bulk specimens 3D-printed from austenitic 316L stainless steel. The characterization is performed both under quasi-static and dynamic loading conditions, while the influence of the printing orientation (i.e., the orientation of the printed layers of the material with respect to the powder bed plane) is studied. Specimens produced with three different printing orientations were subjected to a set of experiments comprising quasi-static uni-axial compression and dynamic compression using a split Hopkinson pressure bar (SHPB) apparatus at two distinct strain rates to assess the strain rate sensitivity of their mechanical responses. Optical measurements of the deformation processes employing a high-resolution (quasi-static experiments) or a high-speed visible-light camera together with a high-speed thermal imaging camera (dynamic experiments) were utilised. An in-house-developed digital image correlation (DIC) procedure was used to evaluate the displacement fields on the deforming specimens to establish the strains. The stress–strain diagrams evaluated from the experimental data together with the thermograms of the dynamic experiments were used to evaluate the influence of the printing orientation on the strain rate dependent compressive characteristics of the bulk 3D-printed 316L stainless steel.

## 2. Materials and Methods

### 2.1. Specimens

To assess the influence of the printing orientation on the compressive behaviour of the investigated material and its strain rate sensitivity, three different sets of specimens with different printing orientations were produced by additive manufacturing of powdered SS316L-0407 austenitic stainless steel. For the 3D-printing procedure, an AM 250 (Renishaw, Wotton-under-Edge, UK) printing device utilising the LPBF technique was used. During the additive manufacturing procedure, 50μm-thick layers of powdered stainless steel with a granularity in a range of 15–45 μm were melted according to the parametric CAD models of the specimens with a chessboard scanning strategy with a 0.1mm hatch distance, 67${}^{\circ }$ increment of rotation angle, 90μs maximum exposure time and 200W maximum power of the laser beam. According to the data provided by the supplier of the printing material, the nominal density of the wrought base material is $\rho_{0}=7.99$ g·cm${}^{-3}$, while the yield strength is 494±14Mpa and 547±3Mpa in the vertical and horizontal directions, respectively.

**Figure 1:**
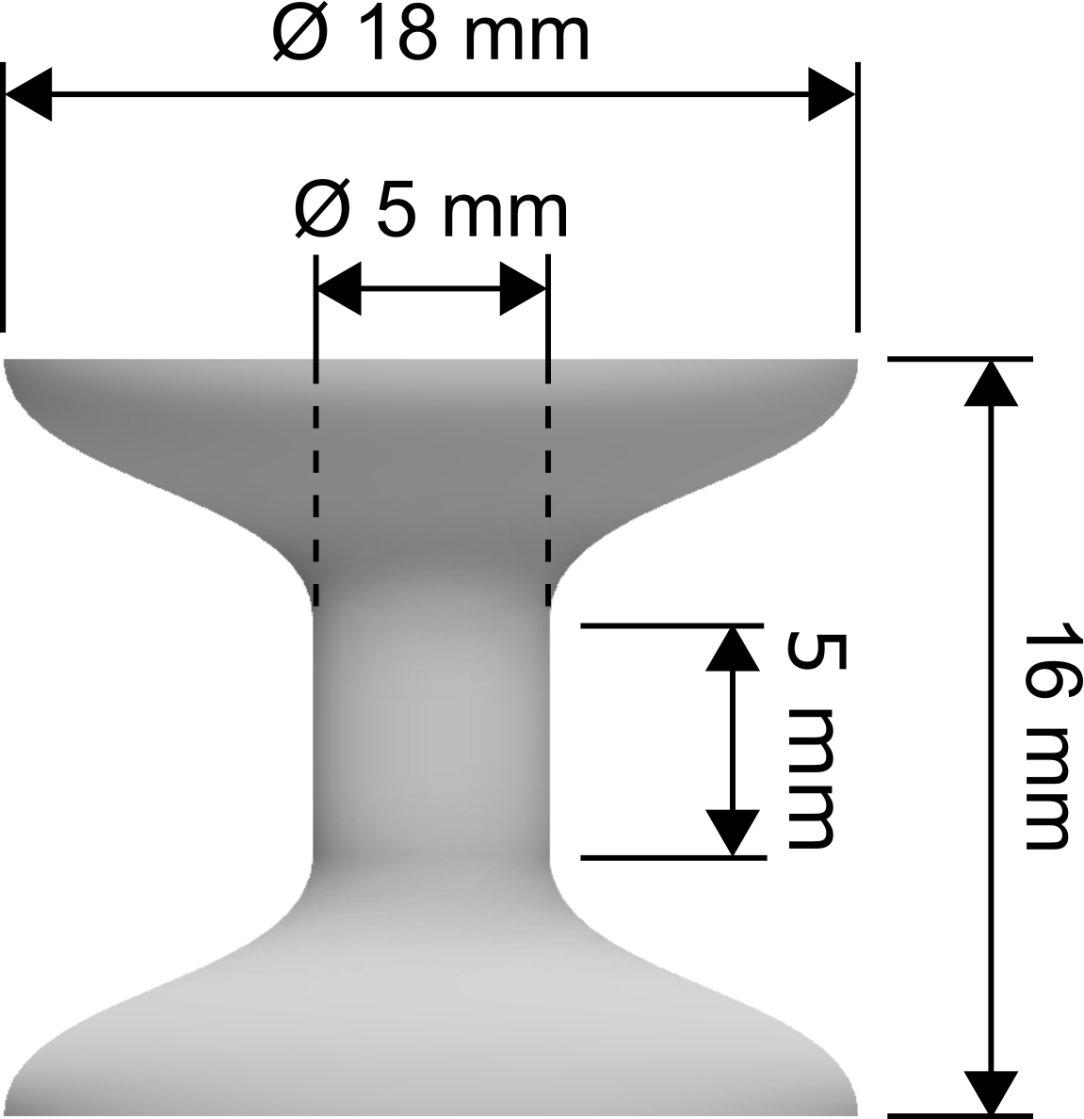
Visualisation of the specimen geometry.

A set of reference cylindrical samples was produced to assess the mass density of the samples considered in the mechanical experiments, yielding ρ=7.52±0.17 g·cm${}^{-3}$. Due to the known properties of the LPBF technique and the production device, it can be assumed that the 6% difference in the mass density is a result of the porosity in the manufactured samples. With respect to the specific conditions during the SHPB compressive testing, a special geometry of the specimens was selected to achieve the required strain rate and overall compressive strain in the sample while preventing localised plastic deformation and damage to the experimental set-up. The resulting sample geometry is of an axisymmetric dog bone-like shape (see Figure 1) with overall dimensions of 18×16mm (greater diameter, overall length) and 5×5mm (lesser diameter, length of the observed region). Furthermore, the greater diameter of the samples at the bar-specimen interfaces enables one to achieve a mechanical impedance similar to the impedance of the bars. Hence, the propagating strain wave is not reflected on the geometrical interfaces and the selected design of the samples, ensuring the effective transfer of the strain wave into and out of the specimen.

**Figure 2:**
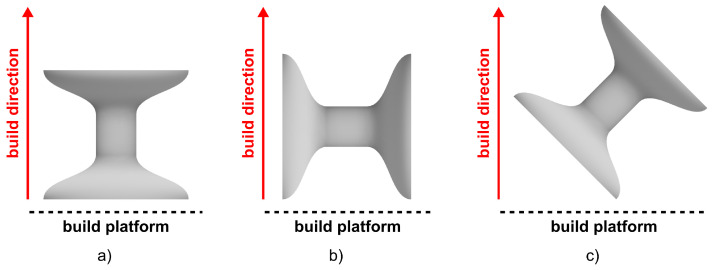
Orientation of the specimens during production: (**a**) vertical ($0{}^{\circ }$ rotation); (**b**) horizontal ($90{}^{\circ }$ rotation); and (**c**) tilted ($45{}^{\circ }$ rotation).

The specimens were 3D-printed in three sets with different orientations with respect to the powder bed plane (see Figure 2). These particular orientations were selected not only owing to their fundamental nature, but also due to the fact that such beam orientations are common in lattice structures including the missing rib structure [28], inverted honeycomb [29], or inverted tetrakaidecahedron [30]. Thus, for reliable interpretation of experiments and the related numerical analyses of common auxetic cellular structures, understanding the characteristics of the base material printed in these orientations is essential.

In total, 21 specimens were investigated in this study, seven from each printing orientation. The outer surfaces of the plates were treated by fine brushing using a bench-top polishing grinder with an automatic specimen mover (Forcipol 202 and Forcimat 52, Metkon Instruments, Osmangazi/Bursa, Turkey). Prior to the compressive experiments, the geometry of the specimens was measured to reveal potential deviations from the CAD prescribed geometry. It was found that, on average, the diameter of the tested area of the specimens was greater by approximately 1.0% for the printing orientation of 0${}^{\circ }$, 2.4% for the direction of $90{}^{\circ }$, and 1.6% for the orientation of $45{}^{\circ }$.

### 2.2. Quasi-Static Experiments

A Model 3382 (Instron, Norwood, MA, USA) electro-mechanical testing device with a maximum load capacity of 100kN was used to assess the quasi-static response of the specimens. The loading rate of the displacement-driven experiments was set to 1mm/min yielding a strain rate of $10^{-3}\;s^{-1}$. The applied force and cross-head displacement were captured at a 10 Hz frequency. A Manta G-504B (AVT, Nuremberg, Germany) high-resolution monochromatic camera with a native resolution of 2452×2056px attached to a TCZR072S (Opto Engineering, Mantua, Italy) bi-telecentric zoom lens was used to observe the deforming samples at a 0.5fps readout rate for the optical evaluation of the displacement and strain fields on the specimens. A KL2500 (Schott, Mainz, Germany) high-power cold-light LED source was used to illuminate the scene.

### 2.3. Dynamic Experiments

**Figure 3:**
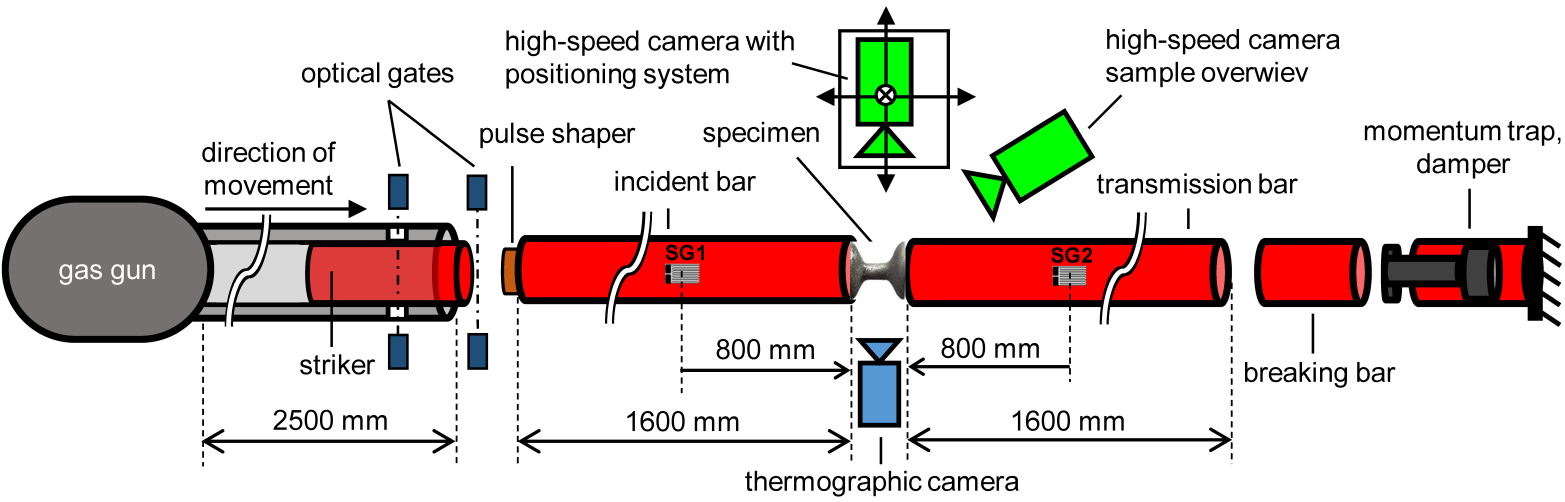
The arrangement of the SHPB experimental set-up: uni-axial compression with aluminium alloy bars.

The high-strain-rate testing was performed in an SHPB (see Figure 3) fitted with measuring bars with a diameter of 20mm and a length of 1600mm. Striker bars with a length of 350mm at a gas-gun pressure of 3.5bar, and 500mm at a gas-gun pressure of 9.5bar were used. The corresponding impact velocities were and 21 m·s${}^{-1}$ and 47 m·s${}^{-1}$, respectively. All the bars were manufactured from a high-strength aluminium alloy (EN-AW-7075), while the incident and transmission bars were supported with low-friction polymer-liner slide bearings with an aluminium alloy housing (Drylin FJUM housing, IGUS, Cologne, Germany) and instrumented with foil strain gauges (3/120 LY61, HBM, Mainz, Germany) in the middle of the bars. At each measurement point, a pair of strain gauges, wired in the Wheatstone half-bridge arrangement, were used. The strain-gauge signals were amplified using a differential low noise amplifier (EL-LNA-2, Elsys AG, Niederrohrdorf, Switzerland) with a gain of 100. The amplified strain-gauge signals were digitised and recorded using a pair of synchronised high-speed 16-bit digitisers (PCI-9826H, ADLINK Technology, Taoyuan City, Taiwan) at a sampling rate of 20MHz. A pair of short-reaction time through-beam photoelectric sensors (FS/FE 10-RL-PS-E4, Sensopart, Wieden, Germany) was placed at the end of the barrel to determine the speed of the striker and served as a trigger for the high-speed camera and strain-gauges data recording. The deformation behaviour of the specimens was simultaneously observed by a pair of high-speed cameras (Fastcam SA-Z, Photron, Tokyo, Japan) and a high-speed thermal imaging camera SC7600 (FLIR Systems, Wilsonville, OR, USA). The main high-speed camera mounted on a motorised positioning system was used for the image acquisition in the direction perpendicular to the axis of the SHPB set-up and its field-of-view comprised the sample itself including the sections of the incident and the transmission bar in contact with the specimen. The region-of-interest involving the deforming specimen was processed by the DIC algorithm to obtain the displacement and strain fields on the specimen surface to evaluate its deformation response at different strain rates. As a main source of optical data in the visible spectrum, this camera was set to a frame rate of 252kfps at a resolution of 256×168px. The other high-speed camera was used for the general sample observation at a higher resolution of 1024×688px at a reduced frame rate of 30kfps. Illumination of the scene was performed using a pair of high intensity LED light sources (Multiled QT, GS Vitec, Bad Soden-Salmünster, Germany).

### 2.4. Digital Image Correlation

The sequences of the visible-spectrum images captured using the high-resolution camera (quasi-static experiments) and the high-speed camera (dynamic experiments) were converted to PNG format using lossless compression and a subsequent DIC analysis was performed to evaluate the displacement fields. DIC is an image processing method based on the tracking of selected correlation points on the surface of the studied object [31]. This method was employed due to the complexity of the specimen geometry and its implementation allowed for the reliable evaluation of the displacements occurring within the test region of the specimen. To obtain the displacement fields for the strain evaluation, a correlation grid containing two lines of tracking points was generated on the observed surface of the specimens.

To perform the DIC analysis, an in-house developed DIC software tool was employed. Our tracking algorithm is based on the template matching technique and uses the sum of squared differences method as a matching criterion. The template matching result shows the level of similarity of the subset image with the source image in a certain pixel position of the source image. The DIC algorithm identifies the position of the subset with subpixel precision. It is achieved by interpolating the template matching results by a third-order bivariate spline and then minimising the interpolated 2D function. For the minimisation of the bivariate spline, the limited-memory Broyden–Fletcher–Goldfarb–Shanno optimisation algorithm [32] was used.

### 2.5. High-Speed Thermography

**Figure 4:**
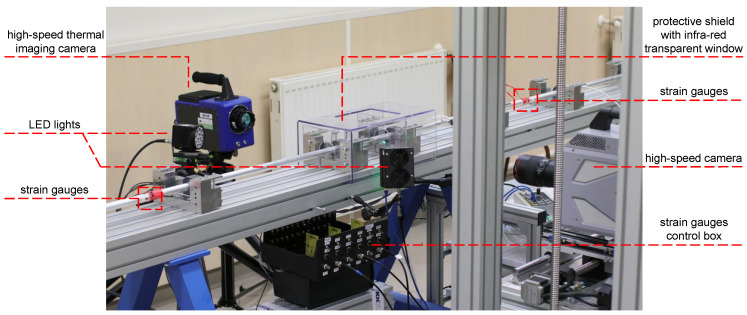
The experimental set-up consisting of the high-speed thermal-imaging camera, the high-speed visible-spectrum camera, and the illumination system.

The dynamic experiments were simultaneously observed by a high-speed thermal imaging camera to evaluate the thermal fields on the surface of the specimens and assess the thermal effects arising from the rapid compression of the samples and to inspect the processes associated with the failure of the specimens. The experimental set-up is shown in Figure 4. An SC 7600 (FLIR Systems, Wilsonville, OR, USA) high-speed thermal imaging camera utilising photon counting InSb focal plane array (FPA) detector operating in the 1.5–5 μm spectral range (SWIR to MWIR band) attached to a 50mmf/2 lens with anti-reflection coated silicon glass optics was used for the thermal imaging. The FPA with a 15μm pixel pitch and a full-frame resolution of 640×512px is actively cooled to enable imaging of low-temperature scenes down to a limit of $-20{\;}^{\circ }C$. In this work, the lens-camera assembly was calibrated for the temperature range of 0–300 °C according to the anticipated thermal response of the samples. To achieve the highest frame rate at a resolution reasonable with respect to the SHPB experiments and the investigated samples, an FPA windowing procedure was employed resulting in 96×44px thermograms acquired at a ≈2 kfps frame rate. During all the dynamic experiments, the thermal imaging was performed through a sapphire (Al_2_O_3_) infrared transparent protective window to guarantee the safety of the thermal imaging optics. To verify the calibration and sensitivity of the high-speed thermal imaging camera, i7 (FLIR Systems, Wilsonville, OR, USA) microbolometric LWIR thermal imager was used.

## 3. Results

For each printing direction, one quasi-static and two sets of three dynamic experiments were performed. Specifically, under the dynamic loading conditions, two different strain rates were investigated. The striker bar, having a length of 350mm, was used in the experiments with a gas-gun pressure of 3.5bar, which resulted in an impact velocity of 21 m·s${}^{-1}$, causing an average strain rate of approximately $1650\;s^{-1}$ (referred to as a low rate later in the text). In the experiments wherein a longer striker bar of length 500mm was accelerated using a gas-gun pressure of 9.5bar, an impact velocity of 47 m·s${}^{-1}$ was achieved, causing an average strain rate of approximately $5100\;s^{-1}$ (referred to as a high rate later in the text). In total, three quasi-static and eighteen dynamic experiments were performed in this study. The optical measurement of the deformation processes employing high-resolution (quasi-static experiments), high-speed visible-light camera, and high-speed thermal-imaging camera (dynamic experiments) were utilised. The displacements of the observed surface of the specimens’ tested areas were evaluated using the DIC method and were used for the calculation of the true stresses and strains.

### 3.1. True Stress–True Strain Analysis

**Figure 5:**
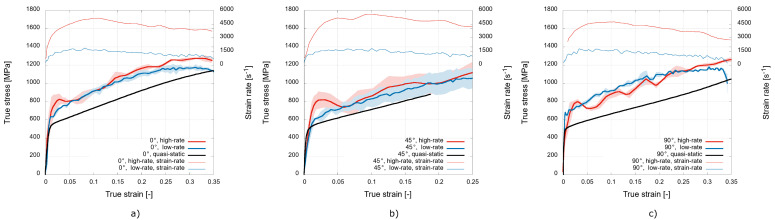
Mean true stress–true strain diagrams: (**a**) 0${}^{\circ }$; (**b**) 45${}^{\circ }$; and (**c**) 90${}^{\circ }$ printing orientations.

**Figure 6:**
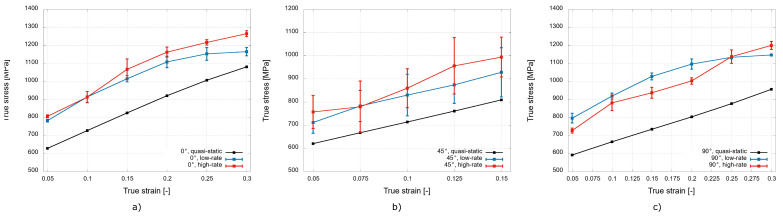
Detailed plastic region of the true stress–true strain diagrams: (**a**) 0${}^{\circ }$; (**b**) 45${}^{\circ }$; and (**c**) 90${}^{\circ }$ printing orientations.

The typical deformation behaviour of the investigated 3D-printed stainless steel samples for the three different printing orientations expressed in terms of the mean true stress-true strain diagrams is shown in Figure 5 together with the average strain rate achieved during the dynamic experiments. The true stress–true strain diagrams reveal the strain rate sensitivity of the mechanical response of the analysed material, as higher stresses were recorded during the dynamic experiments compared with the quasi-static data for all the analysed printing orientations. To quantify this phenomenon, an analysis of the specific values of the stress for a selected strain range in the plastic region was performed. Figure 6 shows the analysed data for particular printing orientations.

For the printing orientation of 0${}^{\circ }$, the average values of the stress in the plastic region increased by approximately 19% during the low-rate experiments and by approximately 25% during the high-rate experiments compared with the quasi-static data. However, the diagram in Figure 6a reveals that more significant differences in the dynamic response occur only for strain values higher than 10%.

The values of the stress in the plastic region also increased for the printing direction of 45${}^{\circ }$ during the transition from the quasi-static to dynamic loading. The average value increased by approximately 16% for the low-rate experiments and by approximately 22% for the high-rate experiments. For this printing direction, the most prominent average difference in the plastic region stresses from the dynamic experiments might be recognised throughout the analysed strain range (see Figure 6b). However, the standard deviations evaluated from the dynamic experiments are relatively large in this case, indicating a significant variety in mechanical response for this printing orientation under the dynamic loading conditions.

For the printing orientation of 90${}^{\circ }$, the average values of the analysed stress in the plastic region increased by approximately 33% during the low rate dynamic loading and by approximately 27% during the high-rate experiments in comparison with the quasi-static loading. Interestingly, the values indicate that the average values of the stress in the plastic region are lower during the high-rate experiments than during the low-rate experiments. A further analysis of the diagram shown in Figure 6c revealed that this phenomenon is more significant for strain values up to 25% and was probably also affected by the oscillatory nature of the stress–strain curve (see Figure 5c) caused by the high strain rate.

**Figure 7:**
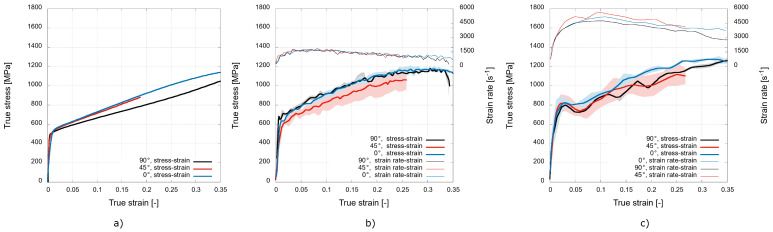
True stress–true strain diagrams evaluated from: (**a**) the quasi-static; (**b**) low rate; and (**c**) high-rate experiments.

To directly compare the deformation behaviour of the material printed with the different printing orientations, diagrams capturing the true stress–true strain diagrams for the particular strain rates are shown in Figure 7.

The quasi-static data shown in Figure 7a reveal a noticeable difference in the mechanical response for the printing direction of 90${}^{\circ }$, where the strain hardening is significantly lower compared to the other printing directions. The diagram in Figure 7b shows the mechanical response during the low-rate experiments, where the most prominent difference in the recorded values of stress is recognised for the printing direction of 45${}^{\circ }$. The stresses in the plastic region are, in this case, approximately 16% lower than in the case of the other printing directions. The mechanical response to the dynamic loading at the high rate in Figure 7c shows the enhanced performance of the 0${}^{\circ }$ printing orientation. The stresses in the plastic region are approximately 9% higher compared to the other printing orientations. The mechanical responses of the 45${}^{\circ }$ and 90${}^{\circ }$ orientations to the high rate loading are rather uniform, as the stress values do not differ to a significant extent. In addition, the oscillatory nature of the stress–strain curve caused by the high strain rate is more prominent for these two directions.

Furthermore, the values of the yield stress for the investigated printing orientations were evaluated from the quasi-static data and are listed in Table 1. The values of the yield stress provided by the supplier of the powdered material (referred to as the reference values further in the text) are 494±14Mpa and 547±3Mpa in the vertical (corresponding to 0${}^{\circ }$ orientation) and horizontal (corresponding to 90${}^{\circ }$ orientation), respectively. The experimentally evaluated value of the yield stress for the printing direction of 0${}^{\circ }$ is approximately 1.9% lower than the reference value. For the printing direction of 90${}^{\circ }$, the value of the yield stress is approximately 2.3% higher. However, when the standard deviations of the reference values are considered, the differences are only 1.4% for the printing direction of 0${}^{\circ }$ and 0% for the direction of 90${}^{\circ }$. The value of the yield stress recorded for the printing orientation of 45${}^{\circ }$ is the lowest compared to the other investigated printing direction and numerically corresponds to the reference value for the 90${}^{\circ }$ orientation.

**Figure 8:**
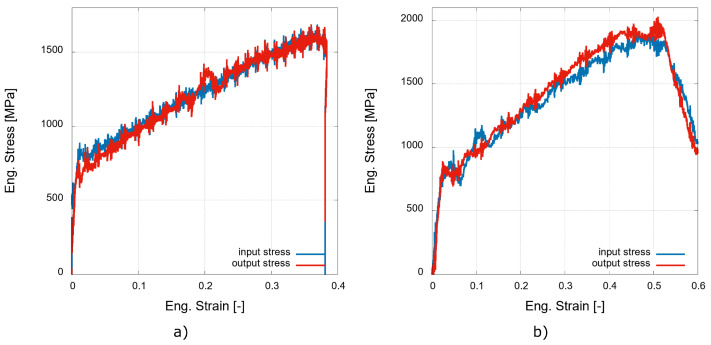
Representative examples of the dynamic stresses equilibrium achieved during (**a**) low-rate and (**b**) high-rate experiments.

An analysis of the dynamic stresses equilibrium was performed for all the experiments to reveal any tendency to non-uniform deformation caused by the strain wave propagation through the specimen. In an ideal case, the stress evaluated at the incident bar (denoted as the input stress) interface quickly converges with the stress evaluated at the transmission bar interface (denoted as the output stress). Representative examples of the dynamic stress equilibrium for the low-rate and high-rate experiments are shown in Figure 8a,b, respectively. The stresses recorded during the low-rate experiment converge at approximately 2% of the strain. On contrary, the diagram for the high-rate experiments shows a very good correlation between the input and output stresses at the specimen-bar interfaces present from the very beginning and during the whole experiment. Generally, the diagrams shown in Figure 8 reveal a good convergence of the dynamic stresses from the initial stages of loading already, and thus, the dynamic data may be considered valid even for the initial values of the strain. This fact enables one to evaluate the values of the dynamic yield stress to assess the strain rate effect on the yield strength of the material.

The values of the yield stress based on an offset of 1% were evaluated from the quasi-static as well as dynamic experimental data and are listed in Table 2. The offset of 1% was chosen to assure the reliability of the data acquired from the dynamic experiments based on the stress equilibrium analysis (see Figure 8) as a good convergence of the input and output stresses may be considered for 1% of the plastic strain.

**Figure 9:**
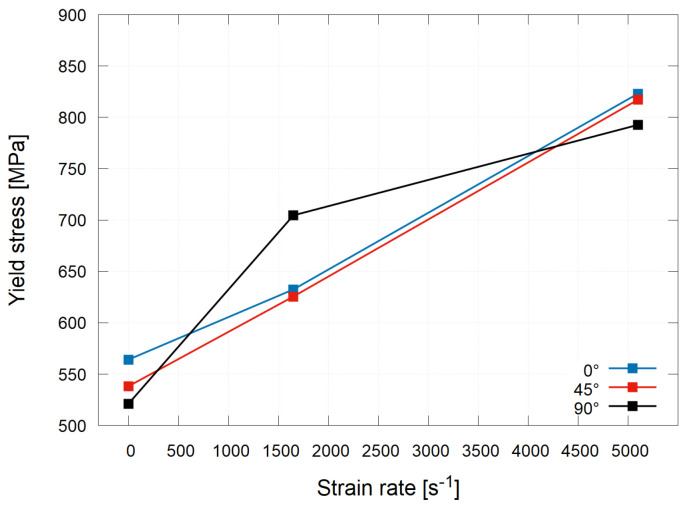
Dependency of the yield stress $R_{p1.0}$ on the strain rate.

Furthermore, the dependency of the yield stress on the strain rate is illustrated in Figure 9. For all the investigated printing orientations, a positive strain rate sensitivity was revealed. The most prominent increase in the value of the yield stress between the quasi-static and dynamic loading was recorded for the specimens with the 90${}^{\circ }$ orientation during the low-rate experiments. However, a complete opposite trend was revealed from the high-rate experiments, where the value of the yield stress for this orientation was the smallest compared to the other investigated orientations. The largest yield stress at the quasi-static loading conditions was recognised for the 0${}^{\circ }$ orientation. Furthermore, a linear dependency of the yield stress on the strain rate was identified for the 45${}^{\circ }$ orientation.

### 3.2. High-Speed Thermography

A high-speed thermal imaging camera was used for the investigation of the thermal fields on the specimens subjected to dynamic compression using the SHPB device that were simultaneously observed by a high-speed visible-light camera. Despite the limited field-of-view of the lenses used in the thermal imaging procedure and the sensor windowing necessary to obtain the highest possible frame rate, it was possible to capture the deforming samples before the rigid body motion of the specimens in the later stages of the experiment caused its displacement beyond the observed area. The characteristics of the thermal imaging camera did not allow one to increase the frame-rate over 2kfps even in the sensor windowing regime, but such a frame-rate was still sufficient to capture a series of four thermograms of the deforming sample in the case of the low-rate experiments and a single thermogram in the case of the high-rate experiments. In all the cases, the high-speed thermal imaging camera was calibrated to the surface emissivity of the samples in the as-delivered state, which was verified using the comparative LWIR thermal imager. According to the mechanical response of the specimens, the thermographical results can be divided into two groups. Here, the first group comprises experiments, where the initial stages of compression in the SHPB apparatus were achieved without the dissintegration of the specimen due to the formation of macroscopic cracks originating from defects along the shear bands in the deforming samples, while the other group comprises the contrary, i.e., specimens damaged early after the arrival of the first incident wave. Overall, one of the three experiments with the specimens having the 0${}^{\circ }$ and 45${}^{\circ }$ orientations resulted in the early development of macroscopic cracks during the low-rate experiments, while the increase in the strain rate to the high rate was required in the case of the 90${}^{\circ }$ specimens to achieve such a mechanical response.

**Figure 10:**
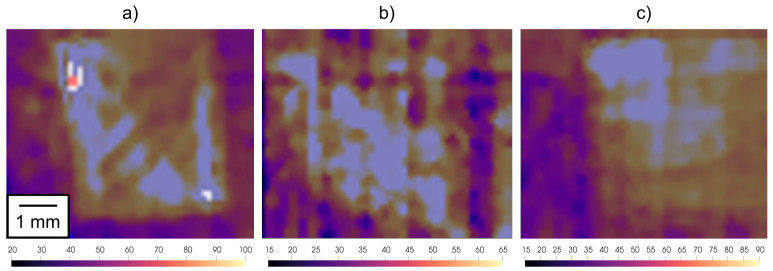
Thermograms captured in the initial stages of the compression for all the orientations of the specimens: (**a**) 0${}^{\circ }$, (**b**) 45${}^{\circ }$, and (**c**) 90${}^{\circ }$.

Figure 10 depicts the representative thermograms captured during the deformation of the specimens with all three orientations.

From the evaluation of the thermal fields that had not dissintegrated in the early stages of the experiments, the highest average surface temperatures of ≈100 °C were assessed in case of the 0${}^{\circ }$ oriented specimens, while ≈$80{\;}^{\circ }C$ and ≈$72.5{\;}^{\circ }C$ were assessed for the 45${}^{\circ }$ and 90${}^{\circ }$ oriented specimens, respectively. The comparison of the series of thermograms from the individual experiments also shows differences in the homogeneity of the thermal fields, where the highest variability was observed in the case of the 45${}^{\circ }$ oriented specimens. This effect cannot be attributed solely to the imaging noise, but rather the surface quality of the specimens as a significantly higher density of supporting pillars had to be used during the printing of the tilted specimens. This resulted in the increased amount of surface imperfections, while also affecting the surface roughness, but the higher density of the supportive pillars was necessary to ensure the stability of the specimens during the printing procedure and to deal with the dissipation of the heat induced by the laser beam melting the powdered material used for the production of the samples.

**Figure 11:**
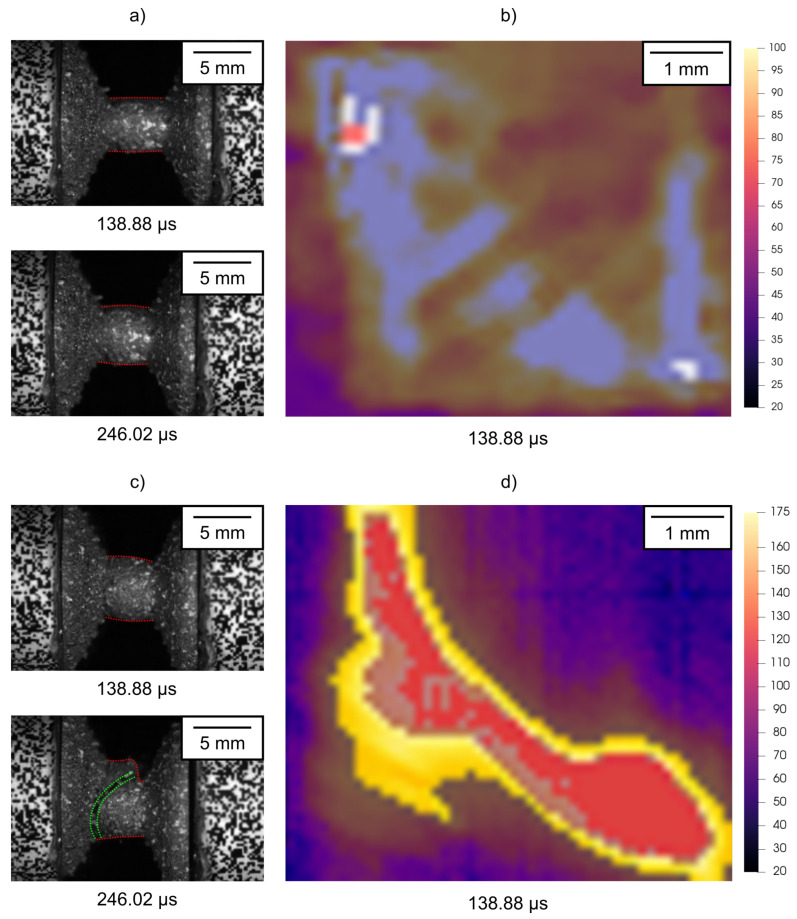
Different behaviour of the specimen with a 0${}^{\circ }$ degree orientation showing the thermograms (**b**,**d**) and visible-light images (**a**,**c**) captured at 138.88μs together with the visible light images captured at 246.02μs revealing the different thermal response of the samples in dependence on the damage evolution.

Figure 11 shows a comparison of the thermograms and the corresponding visible-light images of the experiments from both groups of assessed results in terms of the specimen failures.

**Figure 12:**
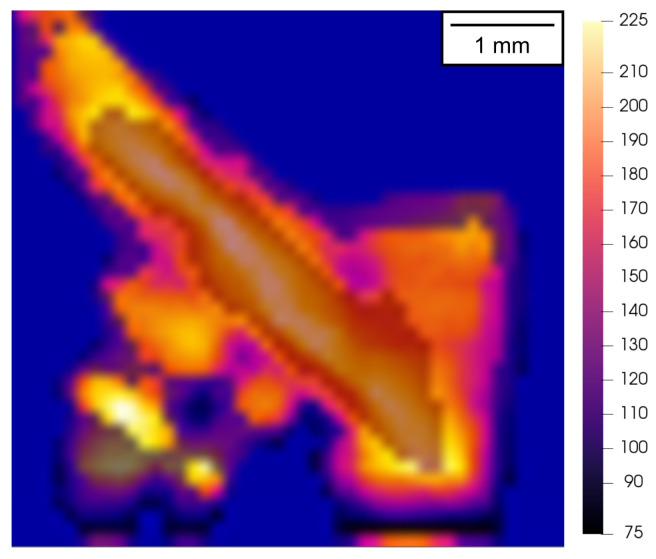
Thermogram of the 90${}^{\circ }$ degree orientated specimen compressed at a high rate (i.e., $5100\;s^{-1}$). The thermogram is segmented to a range of 75–225 °C to highlight the heated regions of the localised deformation, where the macroscopic damage develops during the experiment.

The figure demonstrates two fundamentally different deformation responses of the samples having the same printing orientations. Specifically, the specimen shown in Figure 11a,b exhibits uniform uni-axial deformation coupled with lateral expansion without any axial misalignment of the impact and distal faces, as no macroscopic damage has developed in the presented time frame, yet. In contrast, Figure 11c,d depicts the case where the damage accumulation in the printed specimen resulted in the formation of macroscopic shear damage and the destruction of the sample. Here, the orientation of the crack in the visible and infrared spectrum images is antisymmetric due to the opposite arrangement of the respective cameras and geometry of the defect that diagonally traverses the deforming part of the specimen. It can be seen that the identification of the developing macroscopic crack in the thermograms is possible even before it can reliably be distinguished in the visible-light high-speed camera images. Furthermore, the image segmentation with respect to the temperature can be used as an effective damage development inspection method as shown in Figure 12.

The figure shows a thermogram, where the visualisation was performed with a lower threshold value corresponding to an average temperature of the 90${}^{\circ }$ oriented specimens at the low rate. The segmented temperature range then shows the deformation localised in the shear band, which resulted in the formation of a macrocrack perforating the surface layer of the specimen. However, it has to be noted, here, that the absolute values of the temperature in the segmented areas do not necessarily correspond to the true temperatures. The important factor that has to be taken into account is the change in the emissivity of the observed ROI from the matte-like metal on the surface to the particularly glossy metal in the opened cracks. In this regard, such a measurement has to be considered qualitative in nature, which is, however, sufficient for damage inspection in conditions where visible-light imaging is problematic.

## 4. Discussion

The presented study was focused on the experimental investigation of the strain rate sensitivity of additively manufactured 316L stainless steel and its dependency on three different printing orientations—0${}^{\circ }$, 45${}^{\circ }$, and 90${}^{\circ }$. The results revealed a strong strain rate sensitivity for all the investigated printing orientations, however, the strain rate effect was the most prominent for the 90${}^{\circ }$ orientation, as the values of stress in the plastic region were approximately 30% higher for the dynamic experiments when compared to the quasi-static results for this orientation. Nevertheless, this significant increase is mostly caused by the fact that the specimens tested under quasi-static loading exhibited a notable decrease in the hardening rate (causing lower values of stress in the plastic region) compared with the other investigated printing directions. Such a phenomenon, occurring only during the quasi-static loading, may be explained by the lower coherence of the adjacent layers in the 3D-printed material, which are in the 90${}^{\circ }$ orientation, parallel to the direction of loading. On the contrary, the values of the stress recorded during the dynamic loading of the specimens produced with the 90${}^{\circ }$ printing orientation did not differ from the values obtained for the other investigated printing orientations to any significant extent. Thus, a prominent inertia-related effect occurring under the dynamic loading may be identified for this printing orientation.

Based on the experimental results obtained from the dynamic experiments, the printing orientation of 45${}^{\circ }$ may be considered as the weakest amongst the investigated orientations, as the values of the dynamic stress recorded for this orientation were the lowest compared with the other printing directions. In addition, the dynamic stress values were burdened with the largest deviations compared to the other directions. In general, the printing orientation of 45${}^{\circ }$ generates significant inaccuracies in the geometry of the produced parts due to the relatively more demanding manufacturing process, which also may considerably affect the mechanical response of the investigated specimens. We presume that this effect is the probable source of the highest standard deviations in the mechanical results evaluated from the SHPB measurements for this particular orientation. However, the magnitude of the standard deviation does not exceed the typical errors arising from the nature of the dynamic compression using the SHPB apparatus due to the wave propagation phenomena, particularly the boundary effects on the interface, the differences in the mechanical impedance, and the non-constant strain rate during compression of the investigated specimen (see the typical errors in, e.g., [33]).

Furthermore, the orientation of 0${}^{\circ }$, which has printed layers perpendicular to the direction of loading, was identified as the strongest during both the quasi-static as well as dynamic loading conditions, compared to the other investigated printing directions. However, its tendency toward brittleness, even during low-rate experiments, needs to be well noted.

A tendency to have brittle material behaviour was identified during the dynamic experiments. One of the three experiments at a low rate resulted in the early development of a macroscopic crack for the 0${}^{\circ }$ and 45${}^{\circ }$ orientations. On the contrary, the 90${}^{\circ }$ orientation did not exhibit brittle behaviour during the low-rate experiments. The experiments performed at the high rate led to a rapid macroscopic crack propagation and, finally, the complete disintegration of the specimens in all the cases. The initial cracking of the specimens occurred at a nominal strain of 0.35–0.5 for the 0${}^{\circ }$ and 90${}^{\circ }$ orientations and at a nominal strain of 0.25–0.35 for the 45${}^{\circ }$ orientation.

The experimental results assessed in the presented study indicate the need for further research into the properties of 3D-printed 316L stainless steel at the microstructural level and their effect on the dynamic response of the material. For instance, scanning electron microscopy (SEM) would be beneficial to better understand the changes in the mechanical response of particular printing orientations in relation to fracture characteristics. Furthermore, a combination of hardness measurements and nanoindentation with SEM imaging has the potential to increase the reliability of the formulated conclusions. However, it was not possible to perform such analyses in this study and these topics will be addressed in our future research.

## 5. Conclusions

The experimental investigations presented in this study revealed a significant strain rate sensitivity of the mechanical behaviour of 3D-printed 316L austenitic stainless steel. The compressive deformation response of the specimens produced with three different printing orientations with respect to the powder bed plane was evaluated based on quasi-static and dynamic experiments at two distinct high strain rates. Based on the experimental data, it is possible to conclude:

- Based on comparison of the stress values evaluated from the quasi-static and low-rate dynamic experiments, the most prominent strain rate sensitivity was identified for specimens produced with the printing direction of 90${}^{\circ }$.
- Based on the mechanical response to the dynamic loading, the 0${}^{\circ }$ orientation can be considered as the strongest amongst the investigated printing directions.
- Based on the mechanical response to the dynamic loading, the 45${}^{\circ }$ orientation can be considered as the weakest amongst the investigated printing directions.
- The experimental data from the dynamic experiments for the specimens printed with the 45${}^{\circ }$ orientation were burdened with the largest deviations since such a printing orientation generates the most errors in the geometry of the 3D-printed parts, which significantly affects the evaluated mechanical behaviour for the specimens printed in this manner.
- A tendency of the material to have brittle behaviour was revealed during the dynamic experiments. The 0${}^{\circ }$ and 45${}^{\circ }$ orientations exhibited such behaviour even during the low-rate experiments. The 90${}^{\circ }$ orientation was prone to brittle cracking only during the high-rate experiments.

## Figures and Tables

**Table 1:** Evaluated values of the yield stress for each printing orientation.

Printing Orientation	Yield Stress *R*${}_{p0.2}$
0${}^{\circ }$	536.4Mpa
45${}^{\circ }$	485.7Mpa
90${}^{\circ }$	505.6Mpa

**Table 2:** Values of the quasi-static and dynamic yield stress $R_{p}1.0$ for each printing orientation.

Printing Orientation	Quasi-Static	Low Rate	High Rate
0${}^{\circ }$	564.2Mpa	632.5Mpa	823.1Mpa
45${}^{\circ }$	538.3Mpa	657.6Mpa	817.2Mpa
90${}^{\circ }$	521.7Mpa	704.6Mpa	792.7Mpa

## Data Availability

The data presented in this study are available on request from the corresponding author.
